# Associations between provider communication and personal recovery outcomes

**DOI:** 10.1186/s12888-019-2084-9

**Published:** 2019-03-29

**Authors:** Eunice C. Wong, Rebecca L. Collins, Joshua Breslau, M. Audrey Burnam, Matthew S. Cefalu, Elizabeth Roth

**Affiliations:** RAND Corporation, 1776 Main Street, Santa Monica, CA USA

**Keywords:** Provider communication, Provider respect, Personal recovery, Life satisfaction, Stigma, Empowerment

## Abstract

**Background:**

This study examined whether two types of provider communication considered important to quality of care (i.e., shows respect and explains understandably) are associated with mental health outcomes related to personal recovery (i.e., connectedness, hope, internalized stigma, life satisfaction, and empowerment). This study also tested whether these associations varied by the type of provider seen (i.e., mental health professional versus general medical doctor).

**Methods:**

This sample included participants from the 2014 California Well-Being Survey, a representative survey of California residents with probable mental illness, who had recently obtained mental health services (*N* = 429). Multiple regression was used to test associations between provider communication and personal recovery outcomes and whether these associations were modified by provider type.

**Results:**

Providers showing respect was associated with better outcomes across all five of the personal recovery domains, *connectedness* (β = 1.12; *p* < .001), *hope* (β = 0.72; *p* < .0001), *empowerment* (β = 0.38; *p* < .05), *life satisfaction* (β = 1.10; p < .001) and *internalized stigma* (β = − 0.49; p < .05). Associations between provider showing respect and recovery outcomes were stronger among those who had seen a mental health professional only versus a general medical doctor only.

**Conclusions:**

Respectful communication may result in greater personal recovery from mental health problems. Respecting consumer perspectives is a hallmark feature of both recovery-oriented services and quality care, yet these fields have operated independently of one another. Greater integration between these two areas could significantly improve recovery-oriented mental health outcomes and quality of care.

**Electronic supplementary material:**

The online version of this article (10.1186/s12888-019-2084-9) contains supplementary material, which is available to authorized users.

## Background

Embedded within the World Health Organization’s constitution is the principle that “Health is a state of complete physical, mental and social well-being and not merely the absence of disease or infirmity” [1]. This declaration signifies a move away from a model of mental health that focuses narrowly on clinical symptoms to a broader conceptualization that considers what constitutes and promotes mental well-being. The positive psychology and recovery movements–two forces operating on separate but parallel tracks–have been influential in shifting the mental health field from an exclusive focus on the detection and resolution of disorders to other types of valued goals and outcomes [2].

Originating from within the academic field of psychology, the positive psychology movement is rooted in empirical research and espouses the importance of studying “positive features that make life worth living” for all human beings [3]. Counter to a dominant focus on pathology, positive psychology emphasizes facets of well-being such as life satisfaction, hope, and social connectedness [3, 4]. These dimensions of well-being have been linked to a variety of physical health outcomes including cardiovascular disease, mortality, and longevity, raising their prominence as outcomes to be promoted and tracked at the population level [2, 5]. The recovery movement, in contrast, is a grassroots consumer-driven effort to define personalized goals for recovering from a mental illness that are not limited to clinical outcomes (often referred to as personal recovery) [6–8]. Key dimensions of personal recovery have been primarily derived from qualitative accounts or consensus methods from consumers [4]. A systematic review identified five main personal recovery processes, many overlapping with the domains emphasized in positive psychology, that form the acronym CHIME: connectedness (e.g., social support), hope and optimism, identity (e.g., positive identity, overcoming stigma), meaning in life, and empowerment (e.g., personal responsibility, control over life) [7].

There has been a proliferation of competency statements, standards, and guidelines to aid in the transformation of mental health services to become more recovery-oriented [9–12]. Despite widespread support for recovery-oriented services [11, 13], some have noted that recovery-oriented practice guidelines and provider competences have been created in the absence of a well-developed evidence base [14, 15]. Greater clarification is needed as to what kinds of practices and provider competencies facilitate personal recovery outcomes [15–17]. The dearth of research in this area may be partly due to limitations of existing measures of recovery-oriented practices. A systematic review indicated that measures of recovery-oriented practices have not undergone extensive psychometric testing and do not map onto conceptual frameworks of recovery processes [15, 18].

In contrast, patient-centered care, which shares many of the hallmark features of recovery-oriented services, has been subject to more extensive research and has been an international priority for more than 50 years [19–21]. The Institute of Medicine (now the National Academy of Medicine) defines patient-centered care as: “Providing care that is respectful of, and responsive to, individual patient preferences, needs and values, and ensuring that patient values guide all clinical decisions” [22]. A key feature of patient-centered care involves provider communication that can be easily understood and that demonstrates respect for consumers [20]. Assessing consumer experiences of care including perceptions of provider communication provides essential information on whether patient-centered care is being delivered and is increasingly employed in payment and incentive models for behavioral health organizations [23–27].

To the authors’ knowledge, no study has examined whether consumer experiences of provider communication are linked to personal recovery outcomes and whether this relationship varies by provider type. Using the 2014 California Well-being Survey, a cross-sectional population surveillance survey of California adults with probable mental illness, this study aims to: (1) empirically test whether personal recovery outcomes are related to consumer experiences of two aspects of provider communication – showing respect and explaining things in an understandable way; and (2) assess whether the associations between provider communication and personal recovery outcomes differ depending on whether care is provided by general medical doctors versus mental health professionals. Although consumer experiences have been linked to physical health outcomes [28], it is unclear whether a similar relationship will be found with personal recovery outcomes given the dearth of research in this area. Individuals with a diagnosable mental disorder are more likely to obtain care from general medical providers than psychiatrists or other mental health specialists [29, 30]. Given that care from general medical doctors likely consists of psychotropic prescription medication, whereas mental health professionals may be more likely to provide psychotherapy-based treatment, we hypothesize that the impact of provider communication may be greater for mental health professionals. This is the first study to examine personal recovery outcomes among consumers using a population-based sample in contrast to prior studies that have been primarily conducted with clinic or convenience samples.

## Methods

### Participants and procedures

Individuals who participated in the 2013 California Health Interview Survey (CHIS) and reported symptoms of mental distress were recruited to participate in the 2014 California Well-Being Survey (CWBS). The CHIS is a cross-sectional, random digit-dial telephone health survey (equal proportion land lines and cell phones) that is administered on a continuous basis with a representative sample of California residents [31]. Respondents from the 2013 CHIS who were 18 years or older, completed the CHIS in English or Spanish, consented to be re-contacted for future studies, and scored nine or greater on the Kessler-6 (K6) were eligible to participate in the 2014 CWBS. K6 scores of eight to 12 have been used as a cut-off for mild to moderate distress and greater than 12 for severe distress [32]. The 2014 CWBS respondents were fairly equally distributed between those with mild/moderate (52.8%) and severe distress (47.2%). The CWBS was a telephone survey conducted in English and Spanish between May and August 2014. Informed consent was obtained and study procedures were approved by the authors’ institutional review board. There were 1066 CWBS participants. Response rate was 45.2% [33, 34]. The subset of CWBS participants who reported seeing a general medical doctor (e.g., primary care provider) or mental health professional (e.g., counselor, psychiatrist, social worker) in the past 12 months for a mental, emotional, alcohol or drug use problem (39.3%; *N* = 429) were included in the analytic sample for this study.

### Measures

The CWBS included five measures related to personal recovery: connectedness, hope/personal confidence, empowerment, life satisfaction, and internalized stigma. *Connectedness* was assessed with a two-item scale consisting of the following items: “How often do you get the social and emotional support you need?” (1 = always; 5 = never) [5] and “I have people I can count on” (1 = strongly agree; 5 = strongly disagree) (Pearson correlation = 0.57) [35]. All of the subsequent measures are scored on a 5-point Likert scale (1 = strongly agree; 5 = strongly disagree). *Hope/personal confidence* and *empowerment* were measured using items from the Recovery Assessment Scale (RAS), which taps into several domains of personal recovery [35]. H*ope/personal confidence* included items such as “I can handle what happens in my life” and “Fear doesn’t stop me from living the way I want to.” (alpha =0.84). *Empowerment* was measured using RAS items that comprise the Goal and Success Orientation dimension and included items such as “I have my own plan for how to stay or become well” and “I believe I can meet my current personal goals.” (alpha = 0.78). *Life-satisfaction* was measured with the Satisfaction with Life Scale (alpha = 0.89) [36, 37]. *Internalized stigma* was assessed using the Alienation subscale of the Internalized Stigma of Mental Illness Scale*,* which taps into subjective experiences of not being a full contributing member of society or having a “spoiled identity.” (alpha = 0.80) [38, 39]. Example items include “I feel inferior to others who haven’t had a mental health problem” and “Having had a mental health problem has spoiled my life.” All the personal recovery measures were reverse coded so that higher numbers indicated stronger endorsement of the domain.

*Consumer experiences of provider communication* measures were drawn from The Experiences of Care and Health Outcomes (ECHO) survey [40]. The ECHO is a member of the Consumer Assessment of Healthcare Providers and Systems (CAHPS) family of surveys. Rigorously developed with input from a broad set of stakeholders, it incorporates elements of two other widely used instruments, the Mental Health Statistics Improvement Program Consumer Survey and the Consumer Assessment of Behavioral Health Survey and is approved for accreditation purposes by the National Committee for Quality Assurance. The two items employed by the CWBS both come from the same patient-experience composite, provider communication. Consumer experiences of whether *providers showed respect* were assessed with the question “How often did your provider show respect for what you had to say?”, and consumer perceptions of whether *providers explained things understandably* were assessed with the question “How often did your provider explain things in a way you could understand?” Both items used a 4-point Likert response scale. Responses to these two experience measures were largely bimodal and were dichotomized prior to analysis (0 = Never/Sometimes/Usually; 1 = Always). Participants were instructed to answer the provider communication measures for the provider they saw most recently for a mental health problem.

Control variables included age, gender, race/ethnicity, and psychological distress (i.e., K6 scores).

### Data analysis

Multiple regression models were used to test associations between consumer experiences of provider communication (i.e., showed respect and explained things understandably) and the five personal recovery outcomes (i.e., connectedness, hope/personal confidence, empowerment, life satisfaction, and internalized stigma). Separate models were conducted for each of the personal recovery outcomes. Demographic variables (i.e., gender, age, race/ethnicity) and psychological distress were included as control variables. Main effects for provider communication and provider type seen (i.e., general medical provider only, mental health professional only, or both) were then entered into the model with mental health professional only as the reference group. To assess whether the impact of provider communication on recovery outcomes differed depending on the type of provider seen, a second set of models included separate interactions for each aspect of provider communication by provider type. All analyses were weighted to account for the CHIS sample design and differential nonresponse to both the CHIS and CWBS. Weights incorporated a full sample weight plus 80 replicate weights. Analyses for this paper were generated using SAS/STAT software, Version 9 of the SAS System for Linux.

## Results

Approximately 34.5% (*N* = 141) of respondents reported seeing a mental health professional only, 29.2% (*N* = 91) a general medical doctor only, and 36.3% (*N* = 197) both types of providers in the past 12 months for a mental, emotional, alcohol or drug use problem (see Table 1). Eighty-three percent (*N* = 121) of participants who had seen a mental health professional only and 79% (*N* = 69) who had seen a general medical doctor only reported that their provider had always treated them with respect. Seventy percent (*N* = 98) of those who had sought care from a mental health professional only and 49% (*N* = 47) who had seen a general medical doctor only reported that their provider had always explained things in an understandable way. A significant main effect for provider type was observed such that lower levels of internalized stigma was associated with having seen a general medical provider only compared to a mental health professional only (β = − 0.77; *p* < .001) (see Table 2). Also, having seen both provider types was associated with greater levels of connectedness (β = 0.27; *p* < .05) and lower levels of internalized stigma (β = − 0.55; *p* < .01) compared to a mental health professional only. As seen in Table 2, provider always showing respect was associated with all five recovery outcomes: greater *connectedness* (β = 1.12; *p* < .001), *hope/personal confidence* (β = 0.72; *p* < .0001), *empowerment* (β = 0.38; p < .05), *life satisfaction* (β = 1.10; p < .001) and less *internalized stigma* (β = − 0.49; *p* < .05). These associations correspond to the difference in the outcomes between those who reported that providers always showed respect compared to those who reported that providers did not always show respect. See Additional file 1: Table S1 for adjusted means for personal recovery outcomes for providers always versus did not always show respect by provider type. No statistically or practically significant results were observed for the association between provider always explained understandably and the recovery outcomes except for *internalized stigma* (β = − 0.45; *p* < .05).

**Table 1:** Descriptives of sample, provider type, provider communication, and personal recovery outcomes (*N* = 429)

Variables	Unweighted N	Weighted %
Sociodemographic		
Female	300	65.1
Age		
18–29	49	27.6
30–39	30	14.8
40–49	81	19.1
50–64	201	33.2
65 and up	68	5.4
Race-Ethnicity/Language		
African American	21	9.0
Asian American	9	3.3
Latino-English	56	26.3
Latino-Spanish	16	6.3
White	293	49.9
Other	34	5.2
Provider Type Seen		
General medical doctor only	91	29.2
Mental health professional only	141	34.5
Both	197	36.3
Provider Communication		
Provider Shows Respect		
Never/Sometimes/Usually	80	18
Always	347	82.0
Provider Explains Understandably		
Never/Sometimes/Usually	167	42.0
Always	257	58.0
Personal Recovery Outcomes	Mean	Standard Error
Connectedness	3.8	0.06
Hope/Personal Confidence	3.7	0.08
Empowerment	4.4	0.05
Life Satisfaction	3.2	0.10
Internalized Stigma	2.8	0.11

**Table 2:** Regression models predicting personal recovery outcomes

	Connectedness (*N* = 422)	Hope/Personal Confidence (*N* = 413)	Empowerment (*N* = 418)	Life Satisfaction (*N* = 417)	Internalized Stigma (*N* = 350)
Predictors					
Model 1	*R*^2^ = 0.3053	*R*^2^ = 0.3372	*R*^2^ = 0.2307	*R*^2^ = 0.3585	*R*^2^ = 0.4204
Provider Type ^a^					
Mental health professional only	*Reference*	*Reference*	*Reference*	*Reference*	*Reference*
General medical doctor only	0.01 (.17)	0.20 (.18)	0.00 (.13)	0.42 (.23)	−0.77 (.25)
Saw both provider types	0.52 (.41)	−0.17 (.35)	− 0.05 (.13)	0.47 (.35)	−0.78 (.45)
Provider Communication					
Provider explains understandably	−0.05 (.13)	− 0.10 (.13)	0.09 (.10)	−0.15 (.15)	− 0.45 (.18)
Provider shows respect	1.12 (.21)	0.72 (.16)	0.38 (.13)	1.10 (.18)	−0.49 (.23)
Model 2 ^b^	*R*^2^ = 0.3168	*R*^2^ = 0.3604	*R*^2^ = 0.2569	*R*^2^ = 0.3853	*R*^2^ = 0.4520
Provider Type X Communications Interactions					
General medical doctor only X Explains understandably	−0.15 (.36)	0.40 (.34)	0.05 (.29)	0.70 (.44)	0.11 (.32)
General medical doctor only X Shows respect	−0.56 (.46)	−0.71 (.30)	− 0.64 (.30)	−1.03 (.43)	0.85 (.44)
Both providers X Explains understandably	−0.19 (.23)	0.51 (.34)	−0.16 (.26)	0.46 (.39)	−0.59 (.34)
Both providers X Shows respect	−0.16 (.40)	−0.34 (.35)	− 0.33 (.34)	−0.67 (.36)	0.69 (.47)
Psychological Distress	−0.04 (.01)	−0.06 (.02)	− 0.04 (.01)	−0.06 (.02)	0.1 (.02)
Age	0.03 (.18)	0.26 (.18)	0.14 (.13)	0.27 (.20)	−0.1 (.20)
Female	0.33 (.13)	0.37 (.19)	0.27 (.13)	0.74 (.18)	−0.36 (.17)
Race/Ethnicity Language					
White	*Reference*	*Reference*	*Reference*	*Reference*	*Reference*
African Americans	0.64 (.23)	0.73 (.12)	0.58 (.12)	0.75 (.24)	−0.08 (.28)
Asian Americans	0.47 (.53)	0 (.50)	0.24 (.63)	−0.46 (.51)	1.31 (.36)
Latino English interview	0.14 (.13)	0.14 (.22)	0.17 (.12)	0.11 (.22)	0.3 (.25)
Latino Spanish interview	−0.14 (.25)	0.57 (.36)	0.24 (.30)	0.6 (.26)	−0.21 (1.0)
Other	−0.5 (.32)	0.11 (.21)	−0.13 (.18)	0.01 (.30)	0.73 (.31)

* *p* < .05**; *p* < .01;*** *p* < .001

^a^ Models included controls for age, gender, race/ethnicity, and psychological distress

^b^ Model 2 includes Model 1 variables (which are not presented) plus interactions between provider type and provider communication

The associations between provider always showing respect and all five recovery outcomes were stronger among those who had seen a mental health professional only compared to those who had seen a general medical doctor only, with three of the five interaction terms indicating statistically significant differences in the associations. The associations between showing respect and personal recovery outcomes by provider type (mental health professional vs. general medical doctor) and *p*-values for their differences were: greater *connectedness* (β = 1.36 vs. β = 0.80; *p* = .23), *hope/personal confidence* (β = 1.02 vs. β = 0.31; *p* < .01), *empowerment* (β = 0.72 vs. β = 0.08; *p* < .01), *life satisfaction* (β = 1.63 vs. β = 0.60; *p* < .01) and less *internalized stigma* (β = − 0.97 vs. β = − 0.12; *p* = .06).

With respect to covariates, higher psychological distress and male gender were associated with worse outcomes across all five personal recovery domains. African Americans and Latino Spanish interview participants experienced better personal recovery outcomes, whereas age was not associated with any personal recovery outcomes.

## Discussion

To the authors’ knowledge, this is the first study to empirically test whether consumer experiences with two aspects of provider communication are related to personal recovery outcomes. Individuals who reported that providers always showed respect achieved better outcomes on all five personal recovery domains. Showing respect is a key aspect of patient-centered care and a hallmark of quality mental health care [20]. It has also been identified as a core feature of recovery-oriented services [16]. In an online Delphi survey with consumers, treating consumers with respect was one of the top-rated recovery-related mental health provider competencies [16]. Our results build on this finding suggesting that providers showing respect may be related to consumer recovery outcomes.

Further, our findings indicate that the associations between showing respect and recovery outcomes are even greater for mental health professionals than general medical providers. Given that mental health professionals are more likely to deliver psychotherapy-based treatment, respect may play a more prominent role in facilitating conditions that promote personal recovery outcomes, such as empowerment and hope/confidence, compared to general medical doctors who are often limited to short visits involving psychotropic medication management. Findings are consistent with previous research in which showing respect was the most important dimension of communication associated with overall physician ratings in 23 of 28 medical and surgical specialties [41]. Moreover, showing respect was even more influential in specialty care settings where patients may feel especially vulnerable (e.g., plastic surgery). Showing respect may play a particularly important role in psychotherapy-based treatment where opportunities for more extensive discussion of mental health challenges and associated vulnerability may be greater.

Interestingly, experiences of providers explaining things understandably had no significant associations with any of the personal recovery outcomes except for internalized stigma. Relatedly, providers communicating in an understandable way was not identified as one of the top ranked recovery competencies identified by consumers in the Lakeman (2010) study. Likewise, in a qualitative analysis of recovery-oriented practice guidelines, provider communication was not even mentioned in the four practice domains that emerged, which included promoting citizenship, organizational commitment, working relationship, and supporting personally defined recovery [42]. Supporting personally defined recovery, in which consumers are supported to define their own treatment needs, preferences and goals, is at the heart of recovery-oriented care [4, 7, 42]. Personally defined recovery also dovetails with the concept of shared decision making, a central characteristic of patient- or person-centered care, in which providers and consumers engage in a collaborative partnership to personalize care [43]. Communicating in an understandable manner may be tapping into the conveying of technical treatment information (e.g., psychoeducation), which may not be as directly linked to recovery outcomes. Recovery outcomes may be optimized when providers communicate understandably within the context of supporting personally defined recovery and shared decision making.

This is one of the few studies that have examined the relationship between sociodemographic characteristics and levels of psychological distress to personal recovery outcomes [7]. Our finding that females and certain racial-ethnic minority groups (i.e., African Americans, Latino Spanish interview participants) exhibited better personal recovery outcomes warrant further research. Prior research suggests that the conceptualization of recovery outcomes may differ by gender and race/ethnicity [44, 45]. Likewise, the negative association between psychological distress and personal recovery outcomes suggests the need to better understand how to best facilitate personal recovery outcomes among those with more severe mental illness. For instance, in a small study involving individuals with serious mental illness, interactions with professional staff in which participants felt seen and heard appeared to support personal recovery [46]. Findings should be considered in light of study limitations. We cannot establish whether the associations between provider communication and recovery outcomes are causal given the cross-sectional nature of the study. In addition, the CWBS was designed for population surveillance and could not accommodate full length measures for many of the domains investigated. For instance, the provider communication measures are simple, single-item measures, but were drawn from a validated instrument with strong psychometric properties considered the national standard for evaluating consumer experiences [40]. Further, measures for the personal recovery outcomes of life satisfaction and connectedness were drawn from the positive psychology field, which has developed well-validated measures that have been administered at the population level [5, 36, 37, 47]. The remaining three recovery outcomes (i.e., hope, empowerment, and internalized stigma) were assessed with measures that have been predominantly examined among clinic or convenience samples. The conceptualization and measurement of recovery outcomes continue to be refined and further advancements are needed to facilitate recovery-oriented practices [8, 18, 48]. A key strength of our study is the examination of recovery outcomes with a representative sample of individuals who reported experiencing mental distress and seeing a provider in the past year. Because the sample is not drawn from a particular clinic or provider, the differences by provider type could be examined in a group who saw a diverse sample of providers. It is important to note that our sample excluded certain segments of the population (e.g., homeless, incarcerated, hospitalized) in which the nature and severity of mental illness could significantly differ. Nonetheless, our analyses did control for levels of psychological distress accounting for potential differences in mental illness severity among study participants obtaining services across different mental health care settings. Finally, provider communication overlaps with other areas such as working alliance [49–51] and shared decision-making [52, 53] that have been linked to personal recovery outcomes; additional research is needed to better understand how these consumer experiences relate to one another and personal recovery. Further, some have expressed concerns that consumer experience surveys may tap into providers’ compliance with patient expectations or preferences even when they may be contraindicated (e.g., stopping medication) [54, 55], highlighting the potentially complex relationship between consumer experiences, the delivery of evidence-based care, and personal recovery.

## Conclusion

Recovery-oriented care is increasingly being recognized as a core feature of quality mental health care [21, 56]. Recovery-oriented care embodies many of the same principles of patient-centered care (a hallmark of quality care) and many of the well-being domains within the field of positive psychology. Yet, what constitutes recovery-oriented care both in terms of provider competencies and personal recovery outcomes are still evolving, as are measurable indicators of these domains [16, 42]. Much more extensive work on the measurement of provider behavior and well-being outcomes has been conducted within the areas of quality care and positive psychology. Greater intersections between these currently siloed areas of research could advance the provision of recovery-oriented care [11].

## Additional file

Additional file 1: **Table S1.** Adjusted Means and Standard Deviations for Provider Shows Respect by Provider Type Seen**.** Presents the adjusted means and standard deviations for personal recovery outcomes (i.e., connectedness, hope, empowerment, life satisfaction, internalized stigma) by provider shows respect (i.e., “always” versus “never/sometimes/usually”) and by provider type seen (i.e., general medical doctor only, mental health professional only, and both providers). Means and standard deviations have been adjusted to account for the effects included in the full regression models including covariates, main effects of provider type seen and provider communication, and interactions between provider type and provider communication. (DOCX 14 kb)

## Abbreviations

CAHPS: Consumer Assessment of Healthcare Providers and Systems
CHIS: California Health Interview Survey
CWBS: California Well-Being Survey
ECHO: Experiences of Care and Health Outcomes
