# Supplementary material for: Associations between provider communication and personal recovery outcomes
Source: BMC Psychiatry. 2019 Mar 29;19:102. doi: 10.1186/s12888-019-2084-9 (PMC6439978; doi:10.1186/s12888-019-2084-9)
Supplement: Supplementary file 1 — Table S1. Adjusted Means and Standard Deviations for Provider Shows Respect by Provider Type Seen. Presents the adjusted means and standard deviations for personal recovery outcomes (i.e., connectedness, hope, empowerment, life satisfaction, internalized stigma) by provider shows respect (i.e., “always” versus “never/sometimes/usually”) and by provider type seen (i.e., general medical doctor only, mental health professional only, and both providers). Means and standard deviations have been adjusted to account for the effects included in the full regression models including covariates, main effects of provider type seen and provider communication, and interactions between provider type and provider communication. (DOCX 14 kb) [file 12888_2019_2084_MOESM1_ESM.docx]

**Additional file 1: Table S1**

**Adjusted Means and Standard Deviations for Personal Recovery Outcomes by Provider Shows Respect (Always vs. Never/Sometimes/Usually) and by Provider Type Seen**

|  | **General Medical Doctor Only** | |  | **Mental Health Professional Only** | |  | **Both Providers** | |
| --- | --- | --- | --- | --- | --- | --- | --- | --- |
| **Personal Recovery** | **Always** | **Never/Sometimes**  **/Usually** |  | **Always** | **Never/Sometimes**  **/Usually** |  | **Always** | **Never/Sometimes**  **/Usually** |
| Connectedness | 3.86 (.018) | 3.06 (.184) |  | 3.96 (.007) | 2.6 (.046) |  | 4.21 (.007) | 3.01 (.112) |
| Hope/Personal Confidence | 3.90 (.020) | 3.59 (.032) |  | 3.90 (.021) | 2.87 (.048) |  | 3.69 (.025) | 3.00 (.078) |
| Empowerment | 4.41 (.015) | 4.33 (.028) |  | 4.54 (.004) | 3.82 (.074) |  | 4.43 (.014) | 4.04 (.031) |
| Life Satisfaction | 4.41 (.015) | 4.33 (.028) |  | 4.54 (.004) | 3.82 (.074) |  | 3.43 (.019) | 2.47 (.049) |
| Internalized Stigma | 2.42 (.026) | 2.54 (.085) |  | 2.98 (.042) | 3.95 (.035) |  | 2.54 (.019) | 2.82 (.172) |

Note: Means and standard deviations have been adjusted to account for the effects included in the full regression models including covariates, main effects of provider type seen and provider communication, and interactions between provider type and provider communication.
